# Role of Computer-Aided Drug Design in Drug Development

**DOI:** 10.3390/molecules28207160

**Published:** 2023-10-19

**Authors:** Ruoxu Gu, Fengxu Wu, Zunnan Huang

**Affiliations:** 1 School of Life Sciences and Biotechnology, Shanghai Jiao Tong University, Shanghai 200240, China; 2 Hubei Key Laboratory of Wudang Local Chinese Medicine Research, School of Pharmaceutical Sciences, Hubei University of Medicine, Shiyan 442000, China; 3 Key Laboratory of Big Data Mining and Precision Drug Design of Guangdong Medical University, Key Laboratory of Computer-Aided Drug Design of Dongguan City, Key Laboratory for Research and Development of Natural Drugs of Guangdong Province, School of Pharmacy, Guangdong Medical University, Dongguan 523808, China

The introduction of computational techniques to pharmaceutical chemistry and molecular biology in the 20th century has changed the way people develop drugs. The computer-aided drug design method utilizes the 3D structures of potential drug targets (e.g., enzymes, receptors or ion channels) that are involved in the process of disease and the assembly of potential candidates to design novel molecules or modify existing agonists/antagonists against the target specifically [1,2]. As the development of bioinformatics, computational algorithms are designed to screen biological macromolecules that can be used as targets for the treatment of specific diseases from massive experimental data [3,4]. Moreover, the progress of machine learning and artificial intelligence techniques in the past decade accelerated the field significantly by a smart use of existing data to predict therapeutic targets and ligand-receptor affinities, generate potential candidates for experimental validation and repurpose existing drugs [5,6]. At present, computational techniques have been involved in all stages of drug development, from leading compound screening to preclinical trials. In order to enable more researchers and readers to understand the cutting-edge content in this field, we launched this Special Issue in *Molecules* entitled “*Role of Computer Aided Drug Design in Drug Development*” to present the most recent progress in this field. This Issue includes nine articles in total (two reviews and seven research articles), covering the research field of biomarker and ligand–protein interaction prediction, drug repurposing, ligand binding site identification and largescale virtual screening.

As previously mentioned, computer-aided drug design is an interdisciplinary field that involves a variety of techniques. The two review articles in this Special Issue provide us with a detailed introduction to commonly used methods and tools in drug design. Hasan et al. [7] not only provided a brief introduction about mathematical models in finding novel targets and small molecules but also comprehensively reviewed state-of-the-art structure- and ligand-based computational methods, including QSAR, docking, free energy calculation, ADMET prediction and druggability assessment used for drug design. For structure-based drug design, it is important to identify a potential target and the ligand binding site in the target, so that virtual screening and design are possible. Liao et al. [8] comprehensively summarized the methods, software and databases that are used for possible binding site identification and druggability assessment. Multiple application examples are provided to compare the predictive accuracy and advantages of these tools. A guideline was also proposed for how to select proper methods based on the available data. This review is a good reference for researchers to understand the methods and steps of binding site identification.

Four out of seven research articles in this Special Issue present case studies of virtual screening and drug design using structure- and ligand-based computational methods. Rayevsky et al. [9] virtually screened possible selective ACE2-chemical probes from 3.2 million compounds with the aim of designing small molecules that can be used to study SARS-CoV2-induced neurological disorders. They constructed a novel pharmacophore model using QSAR and docking strategies and then evaluated the activity of the screened ligands using in vitro experiments. Two novel chemotypes were finally discovered that can be used for further study. Another global epidemic that has attracted people’s attention in the past few years is the Monkeypox. Lam et al. [10] employed docking and molecular dynamics simulations to explore the interactions between multiple FDA-approved drugs and five viral Monkeypox proteins. Their drug repurposing studies identified eight drugs that could potentially be used for the treatment of Monkeypox. These two works are examples of how computer-aided drug design techniques improve our ability to manage public health risks. In addition to drug screening and repurposing, molecular modeling is also able to reveal the mechanism of how small molecules regulate the structure and function of macromolecules. Yin et al. [11] combined structure-based techniques, such as molecular docking, and ligand-based techniques, such as pharmacophore modelling, to search for reversible inhibitors targeting the substrate-binding pocket of histone lysine-specific demethylase 1 (LSD1), a treatment target of multiple tumors. Four hit compounds were finally screened out from over two million molecules after drug-likeness evaluation, ADMET screening, MD simulation and binding free energy-based screening. This study is a typical case of the comprehensive application of drug molecular design methods and helps us gain a deeper understanding of the binding mechanisms between compounds and proteins. In another example, Chen et al. [12] performed molecular docking, MD simulations and free energy calculations to study the interactions of two agonists with a wild type and a mutant of the GluN1 subunit of the N-methyl-D-aspartate receptor (NMDAR), a glutamate-gated ionotropic receptor, and elucidated the impact of the mutation on the function of this GluN1 subunit and its associated endogenous ligand affinity. This study is very helpful in providing insight into the design of ligands targeting the mutant of the therapeutic proteins.

The impressive progress of machine learning and artificial intelligence in the past decade is reshaping the field of rational drug design. Another three works in this Special Issue show how data-driven techniques accelerate drug discovery. Li et al. [13] developed a computational method based on graph attention networks and supporting vector machines to predict the association between miRNA and disease. Their work will aid in the discovery of novel biomarkers and therapeutic targets. Although structure-based methods such as molecular docking significantly accelerated drug screening, it is still time- and resource-consuming for screening databases containing millions of molecules. In this regard, machine learning methods used for ligand–receptor interaction prediction have attracted increasing attention. Zhang et al. [14] developed a convolutional network graph-based method, named DeepBindGCN, for ligand–protein binding modes and binding affinity predictions. The model utilized atoms of the residues from the binding pocket and ligands as the nodes and the neighboring information as edges to represent the interaction information. The evaluation of the model is good and has been used to screen lead compounds over two cancer-related therapeutic targets, including TIPE3, a phosphoinositide transfer protein, and the dimer of PD-L1, a critical immune checkpoint ligand and transmembrane protein. It has been revealed that DeepBindGCN is a rapid and reliable tool in hybrid, large-scale screening pipeline.

Besides, Liu et al.’s work [15] provides an application example of drug repurposing using data-driven methods. Specifically, their work constructed a convolutional neural network model to search for potential ligands targeting Parkinson’s disease-associated proteins (LProts) among existing drugs. They also assessed the screened ligands by docking them to several LProts. Their work serves as a typical case for the application of data-driven methods in drug screening.

To summarize, this Special Issue covers most of the computer-aided drug design techniques. It provides the readers with an overview of not only the application of structure- and ligand-based methods for drug discovery but also the development of machine learning methods for the prediction of biomarkers and potential ligands. We sincerely hope the articles in this Special Issue will draw researchers’ attention to the field of rational drug design and prompt the development of novel computational methods for drug discovery.
